# The English of Medical Writers

**Published:** 1916-06-17

**Authors:** C. Thackray Parsons


### THE EDITOR'S LETTER-BOX.

### The English of Medical Writers.

*To the Editor of* The Hospital.

Sir,—Dr. Mercier disagrees with your statement that the Englishman who uses the passive construction with the dative as the subject will find a goodly company to support him. This construction is, however, common in the everyday speech of Englishmen. Though condemned by most, it is recognised as permissible by all English grammarians who give the object of the passive verb a special name, calling it the residual or retained object. The construction does not occur, I believe, in the Authorised Version of the Bible. If it does I shall be grateful to anyone who will give me the reference. But Shakespeare uses it. I can give two examples. In "A Winter's Tale," Act iv., scene 3, Mopsa says, "I was promised them against the feast"; in "Henry VI." Part 2, Act ii., scene 4, Sir John Stanley says, "So am I given in charge, may't please your grace." I will quote two more taken from Murray's Dictionary. Bryce, in "The American Commonwealth," writes: "The Mayor is given power and opportunity"; and Macaulay, in his "History of England," "One of the ringleaders was offered a pardon." The construction is an example of the freedom of the English tongue—a freedom which is the despair of the rigid and logical grammarian. It cannot be used in any of the inflected languages, and I think the methodical and disciplined German would be the last person to employ it. It is pleasing to find Dr. Mercier himself breaking one of the grammarians' cherished rules when, speaking of *two* phrases, he asks, "Which is the clear*est*, neatest, and most accurate?"—I am, Sir, yours faithfully, C. Thackray Parsons.

